# Reassessment of the taxonomic *position of* *Iranocypris typhlops* Bruun & Kaiser, 1944 (Actinopterygii, Cyprinidae)

**DOI:** 10.3897/zookeys.374.6617

**Published:** 2014-01-29

**Authors:** Azita Farashi, Mohammad Kaboli, Hamid Reza Rezaei, Mohammad Reza Naghavi, Hassan Rahimian, Brian W. Coad

**Affiliations:** 1 Department of Environmental Sciences, Faculty of Natural Resources, University of Tehran, Iran; 2 Department of Environmental Sciences, Faculty of Natural Resources, Gorgan University, Iran; 3 Department of Agronomy and Plant Breeding, Faculty of Agricultural Sciences, University of Tehran, Iran; 4 Department of Animal Biology, Faculty of Biology, University of Tehran, Iran; 5 Canadian Museum of Nature, Ottawa, Ontario, Canada

**Keywords:** Iranian cave barb, *Iranocypris typhlops*, *Garra*, phylogeny

## Abstract

The Iranian cave barb (*Iranocypris typhlops* Bruun & Kaiser, 1944) is a rare and endemic species of the family Cyprinidae known from a single locality in the Zagros Mountains, western Iran. This species is “Vulnerable” according to the IUCN Red List and is one of the top four threatened freshwater fish species in Iran. Yet, the taxonomic position of *I. typhlops* is uncertain. We examined phylogenetic relationships of this species with other species of the family Cyprinidae based on the mitochondrial cytochrome *b* gene. Our results show that *I. typhlops* is monophyletic and is sister taxon of a cluster formed by *Garra rufa* (Heckel, 1843) and *Garra barreimiae* (Fowler & Steinitz, 1956) within a clade that includes other species of the genus *Garra*. Based on previous molecular and morphological studies, as well as our new results, we recommend that *I. typhlops* should be transferred to the genus *Garra* Hamilton, 1822.

## Introduction

The Iranian cave barb (*Iranocypris typhlops* Bruun & Kaiser, 1944) is a rare and endemic species of the family Cyprinidae in the Zagros Mountains, western Iran (Mahjoorazad and Coad 2009). The distribution of the species seems to be restricted to a single cave. This species is currently recognized as “Vulnerable” according to the IUCN Red List (IUCN 2013). As such, Coad (2000) using 18 criteria that focused on distribution and habitat, found this species to be one of the top four threatened species of freshwater fishes in Iran. Zalaghi (2011) estimated the population size of the species between 353 and 625 individuals.

**Figure 1.**
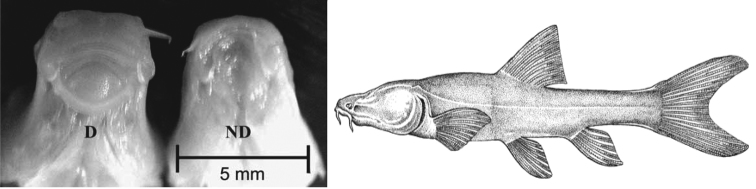
(Left) Ventral view of heads of putative *Iranocypris typhlops*, with (D) and without (ND) a disc, adapted from Sargeran et al. (2008); (Right) *Iranocypris typhlops*, adapted from (Coad 2013).

The species was suggested to be related to the genus *Barbus* Cuvier & Cloquet, 1816 by Bruun and Kaiser (1944) but Saadati (1977) rejected the close relationship with the genus *Barbus*. Coad (2013) proposed that the species may be related to the genus *Garra* Hamilton, 1822. More recently, Hashemzadeh Segherloo et al. (2012) provided the first molecular evidence of the species phylogeny based on the cytochrome *c* oxidase subunit I (COI) gene, which indicated that the species is phylogenetically close to the genus *Garra*. Two sympatric forms have been reported within *Iranocypris typhlops* (Sargeran et al. 2008). They are morphologically distinguished by the presence / absence of a mental disc on the ventral surface of the head (Fig. 1). The mental disc is the lower lip modified into a mental adhesive disc whose posterior margin is discontinuous with the mental region (Zhang 2005). Sargeran et al. (2008) and Hashemzadeh Segherloo et al. (2012) found morphological and molecular differentiations between the two sympatric forms, respectively. Also Hashemzadeh Segherloo et al. (2012) indicated that the two forms might represent separate species based on high intraspecific COI divergence between the two sympatric forms.

Phylogenetic studies of the cavefish populations have shown that some aspects of cavefish systematics are still debated and require molecular analyses to provide evidence on taxonomy and phylogenetic relationships. Detailed molecular studies on some cavefish species have actually shown that their taxonomic position needs a revision based on genetic evidence and that several species, whose description was based on morphological traits only, could be genetically closer to genera different from those to which they are currently assigned to (Romero and Paulson 2001, Colli et al. 2009). In this study, we used the mitochondrial cytochrome *b* (cyt *b*) gene to examine the phylogenetic relationships of the species and in this way to assess the taxonomic position of *Iranocypris typhlops*.

## Study area

**Figure 2.**
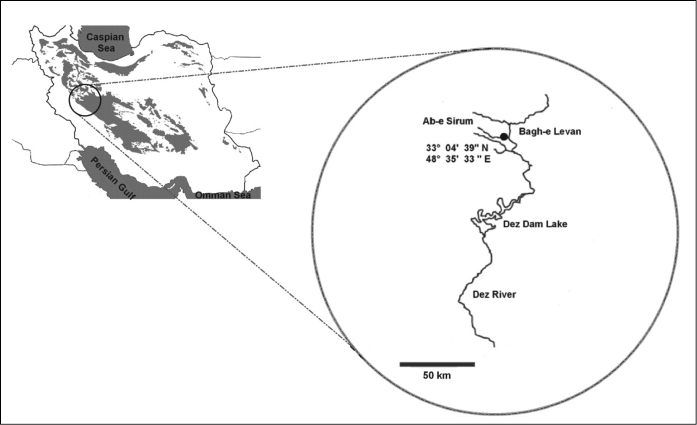
Location of *Iranocypris typhlops* habitat in Bagh-e Levan.

The Iranian cave barb is found in a water cave, the natural outlet of a subterranean limestone system of the Zagros Mountains. The stream below the cave locality is the “Ab-e Serum” which is a tributary of the Dez River, in Lorestan province. The Dez flows into the Karun River, which drains to the head of the Persian Gulf. Further locality details are given in Bruun and Kaiser (1944) and Mahjoorazad and Coad (2009). The cave is located at 33°04'39"N and 48°35'33"E (Fig. 2).

## Methods

Fin-clip samples (approximately 4 mm^2^) of 16 *Iranocypris typhlops* specimens (eight specimens with a mental disc and eight specimens without a mental disc) were collected from the native habitat in November 2012. The fin-clips were stored in 98% ethanol. Total DNA was extracted using the DNeasy-Tissue Kit (Qiagen, Germany) following the manufacturer’s instructions. PCR amplification cyt *b* was performed using primers L15267 (R: 5’-AATGACTTGAAGAACCACCGT-3’) and H16461 (F: 5’-CTTCGGATTACAAGACC-3’) (Briolay et al. 1998). The Polymerase Chain Reaction (PCR) amplification reactions were carried out in 20 µl reaction volume containing: 80 ng of genomic DNA, 1.5 units of Taq polymerase, 1× Roche Taq PCR buffer, 2.5 mM MgCl_2_, 250 µM dNTPs and 0.25 pM of each primer. PCR reactions were conducted under the following conditions: 5 min denaturation at 94 °C; 30 cycles of 40 s at 94 °C, 35 s at 50 °C and 30 s at 72 °C and a final extension at 72 °C for 5 min. PCR products were sequenced on an ABI-3130xl sequencer using the manufacturer’s protocol.

Sequences were aligned using ClustalX (Thompson et al. 1997) and manually checked for inconsistencies. The sequences were deposited in GenBank under accession numbers KF896290 to KF896300. Number of haplotypes, analysis of molecular variance (AMOVA) among and within the two sympatric forms (with and without mental disc), and Kimura two-parameter (K2P) distances (Kimura 1980), were calculated using ARLEQUIN 3.5.1.3 (Excoffier and Lischer 2010).

**Figure 3.**
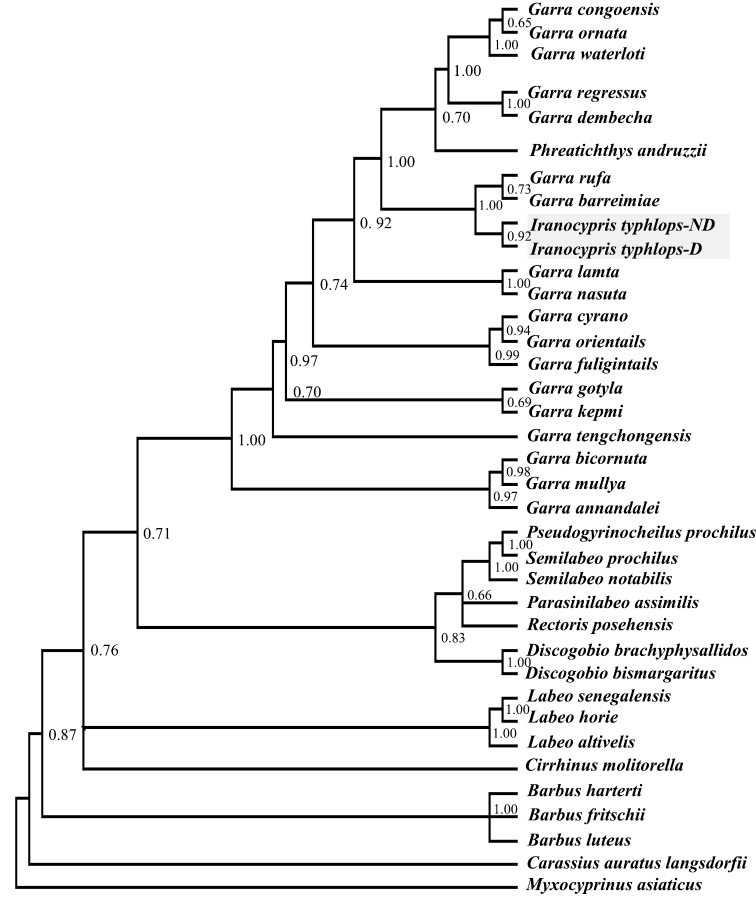
Phylogenetic relationships of *Iranocypris typhlops* based on cyt *b.* The posterior probability values on the branches are the results of BI. *Iranocypris typhlops*-*ND* = specimens without a mental disc, *Iranocypris typhlops*-*D* = specimens with a mental disc.

For the reconstruction of phylogenetic trees, cyt *b* sequences of different cyprinid species (Fig. 3) were retrieved from GenBank and aligned with the sequences of *Iranocypris typhlops*. *Myxocyprinus asiaticus* (Gill, 1878) was included as outgroup (Hashemzadeh Segherloo et al. 2012). Maximum likelihood (ML), neighbor-joining (NJ) and Bayesian analysis (BI) were used to infer phylogenetic trees. The Akaike Information Criterion, the corrected AIC and the Bayesian Information Criterion in jModeltest 2.1.3 (Posada 2008) were used to select an appropriate substitution model of DNA evolution. The GTR model of evolution with gamma shape parameter (G = 1.24) and proportion of invariable positions (I = 0.54) was the selected model according to the three criteria. ML analysis was conducted using PAUP 4.0b10 (Swofford 2003). ML tree searches were performed by heuristic searches. NJ analysis, based on K2P distances, was derived using “dnadist” and “neighbor” executables implemented in Phylip 3.6 (Felsenstein 2005). Support for nodes was assessed by nonparametric bootstrapping (1000 replicates) for ML and NJ analysis and only values > 50% were considered (Colli et al. 2009, Tang et al. 2009). Bayesian analysis was carried out using MrBayes 3.1.2 (Ronquist and Huelsenbeck 2003). The Markov Chain Monte-Carlo (MCMC) search was run for 10^6^ generations, sampling the Markov chain every 100 generations. The first 25% (1000) trees were discarded as burn-in.

## Results

After trimming the alignment, the cyt *b* gene sequences were 904 bp long and 11 haplotypes were found for the 16 sequenced samples (four haplotypes for specimens with mental disc and seven haplotypes for specimens without mental disc). AMOVA showed that 95.72% of the variation in cyt *b* sequences was attributed to differences among the two sympatric forms (Table 1). Also FST value showed significant genetic variation among the two sympatric forms. ML, NJ and BI analyses yielded phylogenetic trees with almost the same topology but the consensus tree of BI supported the relationships among species with higher posterior probabilities (Fig. 3). Genetic divergences among *Iranocypris typhlops* and 22 species of the family Cyprinidae are presented in Table 2.

**Table 1.** Analysis of AMOVA among the two sympatric forms of *Iranocypris typhlops*.

Source of variation	d.f.	Sum of squares	Variance components	Percentage variation
Among sympatric forms	1	143.318	17.815	95.720
Within sympatric forms	14	11.160	0.7971	4.280
Total	15	154.478	18.612	100.000

F_ST_ = 0.957 (P-value = 0.0000)

**Table 2.** K2P distances between *Iranocypris typhlops*, and 22 species of the family Cyprinidae based on cyt *b.*

Species	1	2	3	4	5	6	7	8	9	10	11	12	13	14	15	16	17	18	19	20	21	22	23
*Iranocypris typhlops*-*ND* (1)																							
*Iranocypris typhlops*-*D* (2)	0.041																						
*Garra rufa* (3)	0.069	0.067																					
*Garra barreimiae* (4)	0.086	0.083	0.083																				
*Garra cyrano* (5)	0.119	0.099	0.125	0.131																			
*Garra lamta* (6)	0.127	0.110	0.113	0.128	0.099																		
*Garra congoensis* (7)	0.115	0.110	0.111	0.139	0.122	0.118																	
*Garra nasuta* (8)	0.126	0.116	0.115	0.125	0.114	0.073	0.126																
*Garra fuliginosa* (9)	0.131	0.121	0.133	0.158	0.086	0.101	0.131	0.111															
*Garra ornata* (10)	0.131	0.123	0.121	0.157	0.135	0.128	0.033	0.134	0.143														
*Phreatichthys andruzzii* (11)	0.132	0.125	0.136	0.150	0.117	0.139	0.123	0.131	0.132	0.145													
*Garra orientalis* (12)	0.140	0.124	0.143	0.150	0.067	0.092	0.148	0.125	0.084	0.157	0.145												
*Garra gotyla* (13)	0.143	0.129	0.133	0.160	0.120	0.122	0.135	0.129	0.121	0.151	0.166	0.116											
*Garra regressus* (14)	0.144	0.130	0.133	0.179	0.144	0.126	0.088	0.141	0.155	0.094	0.142	0.161	0.163										
*Garra annandalei* (15)	0.137	0.129	0.132	0.133	0.130	0.093	0.148	0.123	0.113	0.149	0.156	0.104	0.141	0.167									
*Garra dembecha* (16)	0.146	0.132	0.134	0.179	0.146	0.128	0.089	0.143	0.156	0.096	0.143	0.163	0.165	0.001	0.167								
*Garra waterloti* (17)	0.135	0.133	0.121	0.137	0.137	0.144	0.072	0.141	0.149	0.076	0.156	0.153	0.151	0.108	0.153	0.110							
*Garra kempi* (18)	0.155	0.144	0.138	0.156	0.129	0.108	0.158	0.135	0.121	0.165	0.179	0.127	0.132	0.182	0.113	0.184	0.177						
*Garra tengchongensis* (19)	0.163	0.159	0.141	0.171	0.138	0.126	0.143	0.123	0.146	0.156	0.169	0.135	0.144	0.170	0.117	0.172	0.152	0.161					
*Garra bicornuta* (20)	0.188	0.165	0.181	0.179	0.143	0.155	0.165	0.170	0.149	0.163	0.164	0.155	0.159	0.171	0.119	0.171	0.177	0.165	0.176				
*Garra mullya* (21)	0.173	0.162	0.165	0.181	0.149	0.158	0.173	0.162	0.158	0.184	0.181	0.143	0.162	0.206	0.151	0.206	0.173	0.162	0.152	0.146			
*Barbus harterti* (22)	0.195	0.193	0.194	0.209	0.191	0.185	0.177	0.160	0.181	0.192	0.192	0.198	0.196	0.223	0.177	0.223	0.206	0.173	0.186	0.209	0.190		
*Barbus luteus* (23)	0.203	0.193	0.198	0.218	0.195	0.180	0.193	0.175	0.191	0.201	0.197	0.212	0.203	0.233	0.201	0.233	0.226	0.189	0.199	0.244	0.204	0.066	
*Barbus fritschii* (24)	0.200	0.194	0.188	0.189	0.189	0.185	0.179	0.165	0.182	0.190	0.194	0.202	0.190	0.234	0.180	0.234	0.204	0.177	0.176	0.213	0.192	0.029	0.069

*Iranocypris typhlops*-*ND* = specimens without a mental disc, *Iranocypris typhlops*-*D* = specimens with a mental disc.

## Discussion

The phylogenetic trees showed that both forms of *Iranocypris typhlops* form a single clade and that this clade is a sister group of a clade comprising *Garra rufa* (Heckel, 1843) and *Garra barreimiae* (Fowler & Steinitz, 1956). These two sister clades are placed within a large clade that includes the other species of the genus *Garra*, as well as *Phreatichthys andruzzii* (Vinciguerra, 1924). Colli et al. (2009) also surveyed the phylogeny of *Phreatichthys andruzzii* and *Garra barreimiae* with cyt *b* and reported similar results. Therefore, these authors suggested that the taxonomic position of *Phreatichthys andruzzii* should be revised, while earlier Banister (1984) had already reported that based on osteological data the genus *Phreatichthys* is closely related to *Garra*.

Bruun and Kaiser (1944) reported that *Iranocypris typhlops* is similar to the genus *Barbus*. Yet, according to the K2P distances between *Iranocypris typhlops* and species of the genera *Garra* and *Barbus* (Table 2), and the resulting phylogenetic trees, the species is most closely related to the genus *Garra* and only distantly related to the genus *Barbus*. Saadati (1977) rejected a relationship between *Iranocypris typhlops* and *Barbus* species from the Tigris River basin because of their large size differences and lack of a mental disc. Finally, our results are also similar to those of Hashemzadeh Segherloo et al. (2012), who assessed the phylogenetic position of *Iranocypris typhlops* using the mitochondrial COI gene.

We observed a mean K2P divergence of 4.1% between the two forms of *Iranocypris typhlops*. The intraspecific divergence is higher than the mean K2P divergence reported among other fishes, *e.g*. 0.78% for marine fishes (Zhang and Hanner 2012) and 1.1% for freshwater fishes (Hedrick et al. 2006). Hashemzadeh Segherloo et al. (2012) also reported a similar divergence between both forms at COI. The high genetic distances between the two sympatric forms of *Iranocypris typhlops*, along with the morphological differences between the two sympatric forms of *Iranocypris typhlops* (Sargeran et al. 2008), may be due to particularities inherent to evolutionary processes in subterranean habitats (Hashemzadeh Segherloo et al. 2012).

The mental disc is the key character of species of the subfamily Labeoninae including *Iranocypris* and *Garra*. The genus *Garra* is similar to the genus *Iranocypris* in having three rows of pharyngeal teeth (Abbasi and Gharzi 2008, Coad 2013). Conversely, Bruun and Kaiser (1944) described *Iranocypris typhlops* as a new genus and a new species based on its two rows of pharyngeal teeth. Coad (2013) reported one to three teeth in the outer row, three to four teeth in the middle row and three to five teeth in the inner row. This condition, however, is also found in the genus *Garra* (typically 2, 4, 5-5, 4, 2 teeth in each row, respectively). Earlier, Sargeran et al. (2008) investigated morphometric and meristic features of *Iranocypris typhlops* to conclude that this species is similar to species of the genus *Garra*. Finally, we recommend that *Iranocypris typhlops* is transferred to the genus *Garra* and *Iranocypris* Bruun & Kaiser, 1944 is to be regarded as a species of *Garra* Hamilton, 1822.
